# Inhibition of plastid PPase and NTT leads to major changes in starch and tuber formation in potato

**DOI:** 10.1093/jxb/ery051

**Published:** 2018-03-10

**Authors:** Mariette Andersson, Helle Turesson, Stéphanie Arrivault, Youjun Zhang, Ann-Sofie Fält, Alisdair R Fernie, Per Hofvander

**Affiliations:** 1 Department of Plant Breeding, Swedish University of Agricultural Sciences, Alnarp, Sweden; 2 Max Planck Institute of Molecular Plant Physiology, Am Mühlenberg, Potsdam-Golm, Germany

**Keywords:** ADP-glucose, ATP/ADP translocator, plastid, potato, RNAi, *Solanum tuberosum*, soluble inorganic pyrophosphatase, starch, tuber

## Abstract

The importance of a plastidial soluble inorganic pyrophosphatase (psPPase) and an ATP/ADP translocator (NTT) for starch composition and tuber formation in potato (*Solanum tuberosum*) was evaluated by individual and simultaneous down-regulation of the corresponding endogenous genes. Starch and amylose content of the transgenic lines were considerably lower, and granule size substantially smaller, with down-regulation of *StpsPPase* generating the most pronounced effects. Single-gene down-regulation of either *StpsPPase* or *StNTT* resulted in increased tuber numbers per plant and higher fresh weight yield. In contrast, when both genes were inhibited simultaneously, some lines developed only a few, small and distorted tubers. Analysis of metabolites revealed altered amounts of sugar intermediates, and a substantial increase in ADP-glucose content of the *StpsPPase* lines. Increased amounts of intermediates of vitamin C biosynthesis were also observed. This study suggests that hydrolysis of pyrophosphate (PPi) by action of a psPPase is vital for functional starch accumulation in potato tubers and that no additional mechanism for consuming, hydrolysing, or exporting PPi exists in the studied tissue. Additionally, it demonstrates that functional PPi hydrolysis in combination with efficient ATP import is essential for tuber formation and development.

## Teaser

Hydrolysis of soluble plastidial inorganic pyrophosphate, as well as energy import of ATP to the starch-accumulating amyloplast, is essential for starch synthesis and tuber formation in potato.

## Introduction

In addition to its importance as a transient energy store in photosynthetic tissue in plants, starch is also synthesized and stored as insoluble granules in amyloplasts of storage organs, such as tubers and seeds. Starch from storage organs is a major source of energy for humans through consumption, and is also used in a wide range of industrial processes. Research has therefore focused on improving our understanding of the mechanisms behind starch biosynthesis, and potato tubers (*Solanum tuberosum*), being an important storage sink, have been one of the main plant tissues studied. Much focus has centred on the characterization of enzymes participating in the main starch biosynthetic pathway, and studying changes in their enzymatic activities has provided good knowledge of this pathway (Keeling and Myers, 2010; Zeeman *et al.*, 2010; Geigenberger, 2011). Less research has been carried out on some of the auxiliary steps involved in starch formation, such as, for example, energy supply and turnover. In dicots, glucose-6-phosphate is the main hexose phosphate imported into amyloplasts. This transport is mediated by the glucose-6-phosphate/phosphate translocator (GPT) in counter exchange with inorganic phosphate (Pi) (Kammerer *et al.*, 1998). Phosphoglucomutase (PGM) subsequently converts plastidial glucose-6-phosphate to glucose-1-phosphate (Smith and Denyer, 1992), with an ADP-glucose pyrophosphorylase (AGPase) then catalysing the formation of ADP-glucose and pyrophosphate (PPi) from glucose-1-phosphate and ATP (Emes and Neuhaus, 1997). The formation of ADP-glucose is the first committed step in starch biosynthesis, and this metabolite is used as a substrate for granule-bound as well as soluble starch synthases (GBSS and SSS) which polymerize glucose moieties to form the amylose and amylopectin components of starch (Smith *et al.*, 1997). The subcellular localization of AGPase is different in cereal seed endosperm where ADP-glucose formation mainly takes place in the cytosol (Denyer *et al.*, 1996) and is transported into plastids by an ADP-glucose transporter, Brittle-1 (BT1) ( Sullivan *et al.*, 1991; Möhlmann *et al.*, 1998). However, for dicots, plastidial ATP import has been shown to be essential for both ADP-glucose formation and starch synthesis (Geigenberger *et al.*, 2001). The ATP/ADP translocator (NTT) is an antiporter exchanging ATP for ADP across the amyloplast membrane (Heldt, 1969; Schunemann *et al.*, 1993; Tjaden *et al.*, 1998). It has been proposed that, in addition, Pi might act as a third substrate in several transporters of the NTT type (Trentmann *et al.*, 2008). Inhibition of potato *NTT* (*StNTT*) has been shown to cause a decrease in tuber starch content and an increased amylopectin to amylose ratio (Tjaden *et al.*, 1998). Furthermore, this modification resulted in altered tuber morphology with elongated tubers and adventitious budding. In contrast, when *StNTT* was overexpressed, an increase in starch and amylose content in tubers was noted. In agreement with this, Zhang *et al.* (2008) showed that overexpression of Arabidopsis *NTT* (*AtNTT1*) in combination with a pea *GPT* (*PsGPT*) in potato resulted in higher starch content and amylose to amylopectin ratio. This was explained by an increased substrate availability of hexose phosphates and ATP, even though AGPase activity and ADP-glucose levels were themselves unaffected (Zhang *et al.*, 2008).

Although a residual product from sugar nucleotide synthesis, PPi also derives from many other biosynthetic reactions, such as synthesis of nucleic acids, amino acids, and fatty acids. It has been postulated that a low concentration of PPi keeps these reactions in an irreversible state (Kornberg, 1962; Lahti *et al.*, 1988; Sonnewald, 1992; Geigenberger *et al.*, 1998). Cytosolically localized PPi of heterotrophic tissue is consumed in a number of processes including, for example, the sucrose breakdown pathway. In contrast to cytosolic PPi, it is not fully known how the PPi accumulated in plastids is consumed or removed. To date, no PPi-consuming plastidial enzymatic reactions, other than its simple cleavage into two Pi, have been identified. However, a possible energy donor function of PPi in plastids cannot be excluded. It has been speculated that a mechanism for transport of PPi from the amyloplast to the cytosol is present, suggesting that PPi produced from starch metabolism may be exported to the cytosol for participation in sucrose breakdown (Lunn and Douce, 1993; Lara-Nunez and Rodriguez-Sotres, 2004). Another model suggests that PPi pools in amyloplasts and the cytosol are independent of each other (Farré *et al.*, 2006), and that an inorganic pyrophosphatase (PPase) can convert excess PPi in amyloplasts to inorganic phosphate (Pi) for further export. This was based on there being a considerably higher PPase activity in plastids than in the cytosol and, additionally, very low amounts of plastidial PPi compared with total cellular PPi content (Gross and Ap Rees, 1986; Weiner *et al.*, 1987; Farré *et al.*, 2006; Gómez-García *et al.*, 2006). Six soluble forms of *PPase* have been identified in *Arabidopsis thaliana,* where five of them, *AtPPa1–AttPPa5*, are cytosolic enzymes and only one, *AtPPa6*, is localized in the plastid (Schulze *et al.*, 2004). Transient down-regulation of a homologous plastidial *PPa6* gene in *Nicotiana benthamiana* leaves resulted in increased PPi amounts in plastids, confirming plastidial localization of *PPa6* and an absence of a well-functioning chloroplastic PPi exporter in leaf tissue (George *et al.*, 2010). These plants also displayed an alteration in some chloroplast metabolic processes such as starch, chlorophyll, and carotenoid biosynthesis, demonstrating the central role of PPi hydrolysis in many diverse cellular functions. Conversely, the expression of an *Escherichia coli* soluble pyrophosphatase (*EcPPase*) in potato tuber amyloplasts did not lead to any major metabolic changes and the total PPi content was unaffected (Farré *et al.*, 2006).

While PPase and NTT are not directly involved in supplying substrate for building the glucan backbone of the starch granule, they are believed to remove by-products and control supply of energy for starch formation, respectively. In the present study, we investigated the effect of inhibiting a putative plastidial soluble pyrophosphatase gene (*StpsPPase*) and an ADP/ATP transporter gene (*StNTT*; Tjaden *et al.*, 1998) in potato tubers, both independently and in combination. *StpsPPase* inhibition was performed in order to characterize the gene function and to study the effects of a loss of function on starch accumulation in heterotrophic amyloplasts. Even though *StNTT* has been previously characterized (Tjaden *et al.*, 1998), the earlier studies focused on the effect of constitutively and not tuber-specifically down-regulating the gene. Here we studied the consequence of reducing one or both of these genes on starch accumulation and properties, as well as more general aspects of the metabolome and tuber morphology. Our results are discussed in the context of current models of storage starch metabolism in potato tubers.

## Materials and methods

### DNA manipulation

Three RNAi constructs were produced for the individual or simultaneous inhibition of a putative *StpsPPase* and a *StNTT* gene. For the individual inhibitions, fragments of 400 bp of each gene were synthetically produced. For the combined inhibition, 400 bp of each of the two genes were synthetically produced in tandem (Eurofins Genomics). The *AtPPa6* gene (NP196527.1) was used to search publicly available databases, NCBI and TGI, in order to identify a corresponding *S. tuberosum* transcript. The gene fragments represented position 299–699 of the coding region of the putitative *StpsPPase* (XM_006361917.1) and position 1003–1403 of the coding region of *StNTT* (NM_001287865). The selected gene fragments were not found to have homology to other known or putative *S. tuberosum* genes available in the NCBI database. Amino acid sequences of the respective pyrophosphorylase homologues were subjected to sequence alignment (Supplementary Fig. S1 at *JXB* online) using the default settings. A cladogram was created by Tree construction using Neighbor–Joining, Jukes–Cantor, and bootstrapping with 1000 replicates on the CLC Main Workbench 7.9.1 (QIAGEN Bioinformatics). All synthetic fragments were flanked with *attB* sites and cloned as an inverted repeat in the binary vector pGWIWgbss (Hofvander *et al.*, 1992) via pDonor221 (Invitrogen) using Gateway^®^ BP clonase™ enzyme mix and Gateway^®^ LR clonase™ enzyme mix (Invitrogen). The destination vector pGWIWgbss is a modified version of the binary vector pK7GWIWG2 (II) (Karimi *et al.*, 2002), where the 35S promoter has been replaced by a tuber-specific *GBSS* promoter of *S. tuberosum* origin.

### Generation of plant material and growth conditions

The potato cultivar Dinamo was transformed using *Agrobacterium tumefaciens* strain AGL0 harbouring the RNAi constructs. Transformation and regeneration were performed as described previously (Andersson *et al.*, 2003) with the modification that 50 mg l^–1^ kanamycin was used as selection agent. Shoots were confirmed as transgenic by analysis of a small leaf sample using multiplex PCR with two different primer pairs (Sigma Aldrich) to detect T-DNA (nptII) and eliminate shoots with *Agrobacterium* contamination (VirG) (Supplementary Table S1). TheREDExtract-N-Amp Plant PCR Kit (Sigma Aldrich) was used for DNA extraction and PCR amplification. Microtubers were regenerated as described elsewhere (Andersson *et al.*, 2003). Selected lines were cultivated in soil as *in vivo* cuttings in three biological replicates in 7.5 litre pots in controlled greenhouse conditions (16 h day length, 18/15 °C day/night temperature, supplementary light intensity up to ~200 µmol s^–1^ m^–2^ photons, 60% relative humidity) from the end of May to the end of October. Individual tubers from each pot were directly flash frozen in liquid nitrogen at the harvest site.

### Real-time PCR analysis

*In vitro* propagated microtubers and tubers harvested from the greenhouse were frozen in liquid nitrogen. The tissue was homogenized in a mixer mill (MM400, Retsch GmbH), pre-cooled in liquid nitrogen. RNA was extracted with Pure Link RNA extraction reagent (Invitrogen). A 500 ng aliquot of RNA was treated with DNase I (Thermo Scientific) in an 11 µl final volume. For cDNA synthesis, 8 µl of RNA were reverse-transcribed using Superscript III First-Strand Synthesis Supermix for qRT-PCR (Invitrogen). Transcript quantification was performed on a BIO-RAD C1000 Thermal Cycler, CFX 96 Real-Time System using Maxima SYBR Green/ROX qPCR Master Mix (2X) (Thermo Scientific), 1.6 µl of cDNA (1:10 dilution), and 0.3 µM primers (Supplementary Table S1) in a final reaction volume of 20 µl. All PCRs were run in three biological replicates with two or three technical replicates. Melting curve analysis was performed and amplicons were inspected visually on a 1.5% agarose gel.

Normalized expression levels of genes of interest (GOI) were related to expression levels of the reference gene *actin*. Calculations were made according to the equation 2^–∆∆Ct^ where ∆Ct=Ct (GOI)–Ct (actin) and ∆∆Ct is ∆Ct (treated)–∆Ct (untreated).

### Dry matter and starch content determination

Dry matter was measured on tuber tissue weighed before and after 3 d of freeze-drying (Coolsafe, Scanvac). Starch content was determined on homogenized freeze-dried tuber tissue using a Total Starch (AA/AMG) Assay Kit (Megazyme) according to AOAC Method 996.11 and AACC Method 76.13. The samples were pre-treated to wash away free sugars and other solubles by adding 2.5 ml of 80% ethanol to 50 mg of homogenized freeze-dried tuber tissue and incubation at 85 °C for 5 min. A further 2.5 ml of ethanol was added and the samples were centrifuged at 1000 *g* for 10 min. The pellet was re-suspended in 80% ethanol and pelleted again as described above. Subsequent measurements were performed according to the manufacturer’s protocol. Starch content was determined based on a standard curve made using a maize control supplied by the manufacturer.

### Analysis of amylose content

Starch was extracted and the amylose to amylopectin ratios were determined on greenhouse-grown tubers using a spectrophotometric iodine-based method previously described (Andersson *et al.*, 2006).

### Staining of starch granules and tuber tissue

Potato tuber discs of ~0.5 mm thickness were immersed in 50% Lugol’s solution (Scharlau) for 1 min and then rinsed twice in water. The discs were analysed and documented on a light table.

Lugol’s solution with glycerol (1:1) was added to purified starch granules or thin tuber discs. The stained tissue was studied under a light microscope (Leica Microsystems).

### SDS–PAGE of starch-bound proteins

Frozen tubers were homogenized in Retsch MM Mixer mill containers, pre-cooled in liquid nitrogen. Starch was extracted from the obtained homogenate (Larsson *et al.*, 1996). Care was taken to allow the sedimentation of small granules (i.e. the times between each wash step were extended to 2 h or overnight). An extra purification step was applied by filtering through nylon mesh (100 µm). Starch-bound proteins were extracted from 10 mg of starch using 100 µl of extraction buffer (Denyer *et al.*, 1997) of which 20 µl was analysed on an SDS–polyacrylamide gel (10% ClearPAGE, CBS Scientific). The gel was washed three times for 5 min in deionized water and immersed in GelCode Blue Stain reagent (Pierce) for 60 min, and subsequently washed in deionized water until clear bands appeared.

### Metabolite analysis

Hexose phosphates, nucleotide sugars, AMP, and ADP were quantified by reverse-phase LC coupled to tandem MS (LC-MS/MS) analysis, as previously described (Arrivault *et al.*, 2009, 2015), starting from 15 mg FW. ATP was measured by coupled enzymatic assays, taking care to use freshly prepared extracts as described in Arrivault *et al.* (2009). Metabolite profiling of potato tuber samples by GC-MS was performed as described previously (Lisec *et al.*, 2006). Samples were analysed using GC-MS (ChromaTOF software, Pegasus driver 1·61; LECO). The chromatograms and mass spectra were evaluated using TagFinder software (Luedemann *et al.*, 2012). Metabolite identification was manually supervised using the mass spectral and retention index collection of the Golm Metabolome Database (Kopka *et al.*, 2005). Peak heights of the mass fragments were normalized on the basis of the fresh weight of the sample and the added amount of an internal standard (ribitol).

### Enzyme activity analysis

Crude enzyme extracts were prepared from 100–150 mg of tuber tissue as previously described (Jelitto *et al.*, 1992). Before extraction, tubers were homogenized in a Mixer Mill MM400 (Retsch), in pre-cooled containers. Aliquots were snap-frozen in liquid nitrogen and stored at –80 °C. PPase was assayed as previously described, with the modification that the phosphate produced was detected as described by Fusari *et al.* (2006); volumes were adjusted and activities detected in a 96-well plate reader (Multiskan GO, Thermo Scientific). AGPase was assayed according to Jammer *et al.* (2015). All measurements were made in three biological and two technical replicates.

## Results

### Generation of potato lines with tuber-specific reduction of *StpsPPase* and/or *StNTT* expression

By screening publicly available databases (NCBI and TGI), a single putative *S. tuberosum* plastidial soluble pyrophosphatase (StpsPPase) (XM_006361917.1) was identified having 74% protein sequence homology with the plastidial-localized *A. thaliana* PPa6 (AtPPa6). A phylogenetic tree with Arabidopsis *PPase* genes and potato *PPase* homologues shows that the *StpsPPase* has a close relationship with the plastidial-localized *AtPPa6.* It also shows that *StpsPPase* is the only potato *PPase* homologue clustering together with *AtPPa6* (Supplementary Fig. S1). An *StNTT* gene has previously been described and characterized following its constitutive overexpression and down-regulation in potato (Tjaden *et al.*, 1998). In the present study, three different RNAi vector constructs were used to generate potato lines suppressed in *StpsPPase* and *StNTT* expression levels, targeting the genes either individually or simultaneously. For all three constructs, the *GBSS* promoter was used to ensure tissue-specific inhibition of the gene(s). The constructs were introduced into potato by *A. tumefaciens*-mediated transformation of leaf tissue (*S. tuberosum* cv. Dinamo). Regenerated transgenic shoots were propagated in tissue culture, and cuttings were grown to produce *in vitro* microtubers, which were screened for reduced transcript levels of the target genes using real-time PCR. Two lines from each of the single-gene-targeted experiments were selected for further studies. Regenerated lines with the *StpsPPase* and *StNTT* genes targeted simultaneously were, in general, unable to produce microtubers *in vitro*. Therefore, only one line could be selected based on reduced transcript levels of both target genes. In addition, five confirmed transgenic lines were randomly selected among the double-gene-targeted regenerated shoots. In total, 10 lines were propagated under controlled conditions in the greenhouse to obtain fully developed potato tubers for subsequent studies.

### Dry weight, starch content, and composition are severely affected in potato tubers following inhibition of *StpsPPase*

**Fig. 1.**
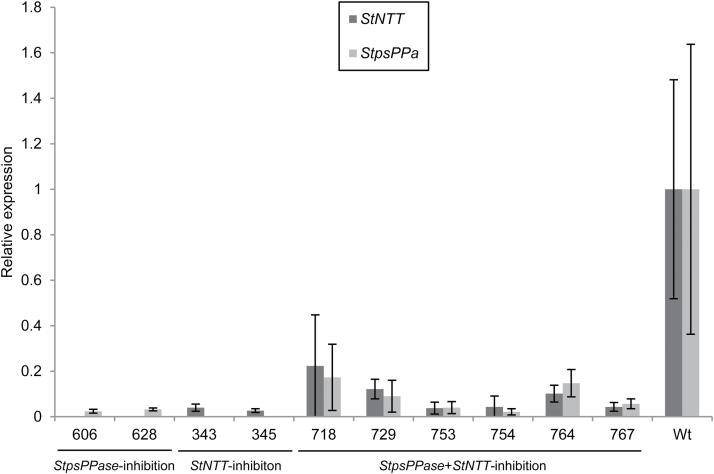
*StpsPPase* or/and *StNTT* expression levels in greenhouse-grown potato tubers of RNAi lines and the wild type (Wt). Expression levels were normalized to the reference gene actin. Expression levels in the Wt were set to 1. Data are mean values (±SD) of three biological replicates.

**Fig. 2.**
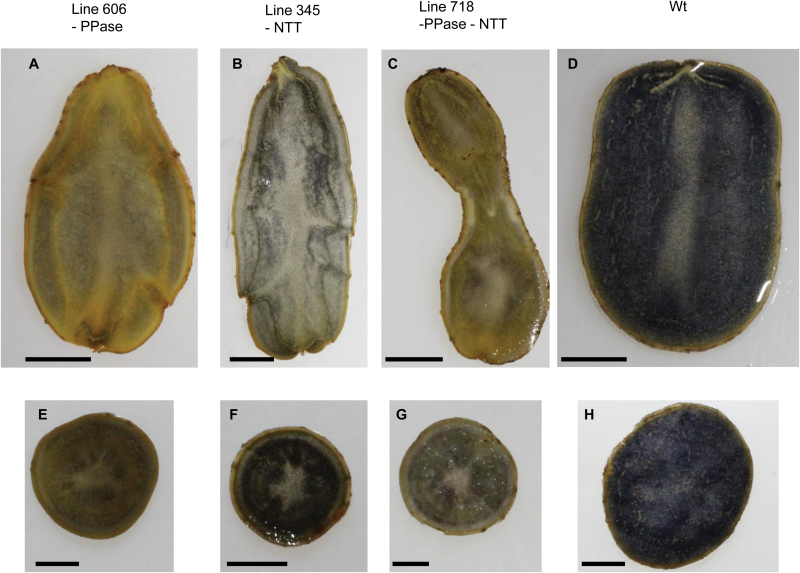
Cross-sections of tubers stained with iodine (Lugol’s solution) to visualize the presence of starch. (A–D) Longitudinal, (E–H) transversal. Lines 606 (*StpsPPase* inhibition) (A, E); 345 (*StNTT* inhibition) (B, F); 718 (*StpsPPase+StNTT* inhibition) (C, G); and the wild type (D, H). Scale bar=1 cm.

**Fig. 3.**
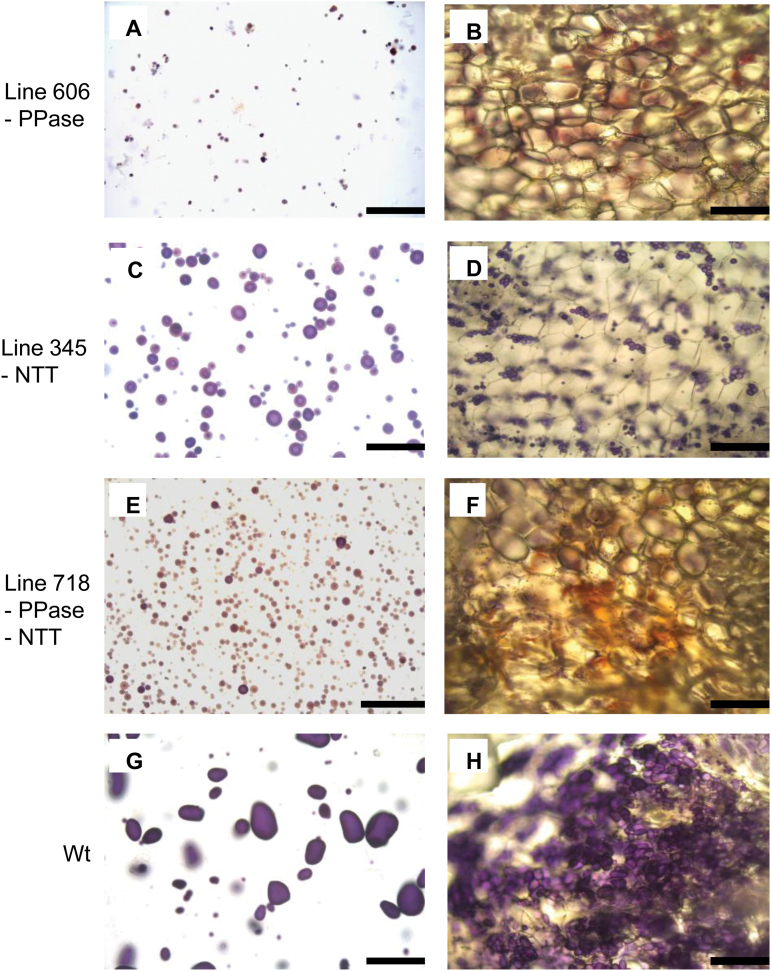
Fresh tuber tissue, homogenized (A, C, E, G) and thin hand-cut (B, D, F, H), stained with iodine solution (Lugol’s or Lugol’s:glycerol) to visualize starch granules. Lines 606 (*StpsPPase* inhibtion) (A, B); 345 (*StNTT* inhibition) (C, D); 718 (*StpsPPase+StNTT* nhibition) (E, F); and the wild type (G, H). Scale bars in (A), (C), (E), (G)=100 µm; in (B), (D), (F), (H)=200 µm.

Two *StpsPPase*-inhibited lines, 606 and 628, were found to lack almost completely any detectable *StpsPPase* gene expression in their tubers (Fig. 1). Tuber dry weight decreased from 27% of FW in the wild type to 10% and 11% of FW in lines 606 and 628, respectively (Table 1). The low dry weight could be explained by a drastic decrease in tuber starch content, dropping from 19% of FW in the wild-type line to 2% of FW in both *StpsPPase*-inhibited lines (Table 1) as determined enzymatically. A low starch content was also confirmed by iodine staining of tuber discs, where high abundance of starch in wild-type tubers was seen as an overall blue-black staining of the discs (Fig. 2, D, H), while only minor staining could be detected on tuber discs from the *StpsPPase*-inhibited lines (Fig. 2A, E). Closer investigation of tuber cells using light microscopy revealed few starch granules in the inhibited tubers (Fig. 3B, H). Examination of starch from homogenized tuber tissue of the transgenic lines revealed a changed starch granule size and shape. In the *StpsPPase*-inhibited tubers, the granules were spherical with a diameter of ~15 µm or less (Fig. 3A), while the majority of the wild-type granules were ovoid, ranging in size from 25 µm to 100 µm (Fig. 3G).

**Table 1.** Yield, starch, and amylose content of greenhouse-propagated *StpsPPase*- and *StNTT*-down-regulated potato tubers

Sample	Inhibited gene(s)	Σ tubers/plant	Σ tuber yield/plant (g)	Average tuber weight (g)	DW (% of FW)	Starch (% of FW)	Amylose (% of total starch)
606	*StpsPPase*	90 ± 14*	730 ± 100*	8.1 ± 0.9*	9.6 ± 0.3*	2.1 ± 0.0*	4.9 ± 0.4*
628	*StpsPPase*	45 ± 32	252 ± 265	5.5 ± 2.2*	10.9 ± 1.2*	2.1 ± 0.7*	2.7 ± 0.1*
343	*StNTT*	40 ± 9*	396 ± 121	10.0 ± 1.3*	13.5 ± 1.8*	6.4 ± 1.1*	8.8 ± 1.6*
345	*StNTT*	62 ± 8*	481 ± 117	7.8 ± 1.0*	15.8 ± 0.6*	7.8 ± 0.5*	5.2 ± 1.0*
718	*StpsPPase*+*StNTT*	28 ± 18	97 ± 114*	3.4 ± 2.1*	9.3 ± 2.1*	1.7 ± 0.5*	2.7 ± 0.1*
729	*StpsPPase*+*StNTT*	10 ± 3	20 ± 8*	2.1 ± 0.2*	10.6 ± 2.3*	2.6 ± 1.1*	3.0 ± 0.5*
753	*StpsPPase*+*StNTT*	10 ± 4	88 ± 27*	8.6 ± 0.4*	13.8 ± 3.0*	6.4 ± 2.6*	3.1 ± 0.3*
754	*StpsPPase*+*StNTT*	34 ± 9*	383 ± 170	11.2 ± 5.8*	9.9 ± 1.3*	2.6 ± 0.5*	3.0 ± 0.1*
764	*StpsPPase*+*StNTT*	10 ± 4	20 ± 23*	2.0 ± 1.6*	9.7 ± 0.3*	1.9 ± 0.3*	3.9 ± 0.4*
767^*a*^	*StpsPPase*+*StNTT*	59 ± 22*	488 ± 282	8.2 ± 4.6*	9.5 ± 1.0*	2.0 ± 0.4*	4.4 ± 0.3*
Wt	–	11 ± 5	346 ± 89	32.4 ± 28	27.4 ± 1.0	18.5 ± 1.2	28.0 ± 0.7

Data are means ±SD: tubers/plant, *n*=3; tuber yield/plant, *n*=3; DW, *n*=9; starch as a percentage of FW, *n*=9; amylose (% of total starch), *n*=9. Results marked with an asterisk differ significantly from the wild type (Wt) using one-way ANOVA, Tukey comparison method (*P*=0.05).

^*a*^ Missing value from one plant)

### *StpsPPase* inhibition leads to alteration in the amylopectin/amylose ratio

Starch composition was analysed using an iodine-based quantitative spectrophotometric method. The *StpsPPase*-inhibited tubers revealed a significant alteration in the amylose to amylopectin ratio where the apparent amylose content decreased from 28% in the wild type to 3% and 5% of total starch in lines 606 and 628, respectively (Table 1). Very low amylose content is commonly associated with low expression or activity of GBSS. To investigate whether the transcription of *GBSS* was affected, the gene expression level and a profile of starch-bound proteins were analysed in line 606, revealing no significant decrease of expression or amount of GBSS in the studied line compared with the wild type (Supplementary Fig. S2A).

### Tuber number and yield are severely affected in *StpsPPase*-inhibited lines

**Fig. 4.**
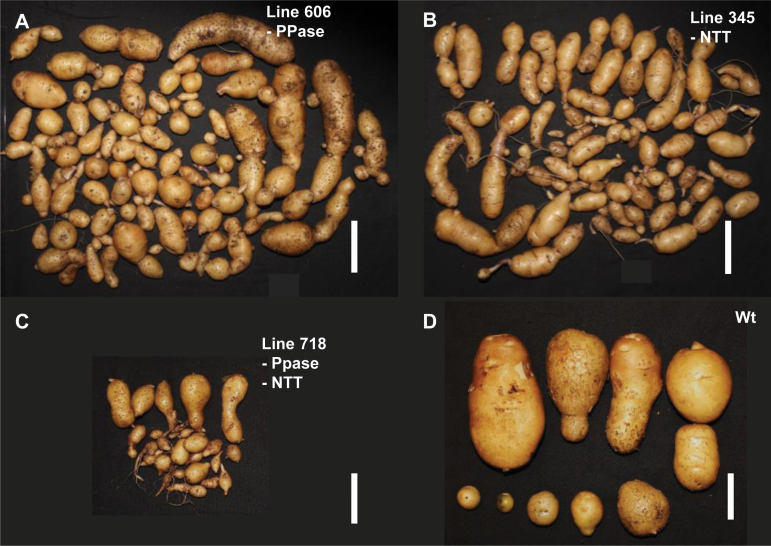
Tubers obtained from one potato plant. (A) line 606 (*StpsPPase* inhibition); (B) line 345 (*StNTT* inhibition); (C) line 718 (*StpsPPase+StNTT* inhibition); and (D) the wild type. Scale bar=5 cm.

The average number of tubers produced per pot was considerably higher in the *StpsPPase*-inhibited lines compared with the wild-type plants (Fig. 4A and D, respectively; Table 1). However, the tuber size was, on average, much smaller in both transgenic lines, being most apparent in line 628, which had an average tuber fresh weight one fifth of the average of that of wild-type tubers (Table 1). In line 606, the number of tubers was nine times higher than in the wild type but, due to smaller tubers, the total tuber mass per plant was only increased 2-fold. In line 628, the number of tubers varied widely between different pots, while the average number of tubers was three times higher compared with the wild type. However, the very small tubers of this line resulted in a total tuber weight per plant that was lower than that for the wild type.

### Tuber-specific inhibition of *StNTT* affects major tuber characteristics, but to a lesser extent than for *StpsPPase* inhibition

Two lines, 343 and 345, with almost complete inhibition of *StNTT* transcripts, were selected for further study (Fig. 1). The inhibition of *StNTT* resulted in a major decrease in tuber dry weight, from 27% to 14% and 16% of FW; tuber starch content also fell from 18% to 6% and 8% of FW compared with the wild type in both inhibited lines, respectively (Table 1). The starch granules from the *StNTT*-inhibited tubers were located close to the cell wall (Fig. 3D), and were spherical and ~15–40 µm in diameter (Fig. 3C), which is about half the size of wild-type granules (Fig. 3G). The amylose content of purified starch from the *StNTT*-inhibited tubers was low, being reduced from 28% in the wild-type line to 5% and 9% of total starch in lines 343 and 345, respectively (Table 1). *GBSS* transcript levels and protein content were not significantly altered in line 343 (Supplementary Fig. S2A). The two *StNTT*-inhibited lines yielded four and six times more tubers than the wild type (Fig. 4B and D, respectively). The tuber yield per plant was higher in both lines, with a 16% and 41% (FW) increase compared with the wild type.

### No additive effect of simultaneous *StpsPPase* and *StNTT* inhibition on dry weight, starch content, and composition

A major decrease in transcript levels of both *StpsPPase* and *StNTT* genes was confirmed in six lines (718, 729, 753, 754, 764, and 767) inhibited for both genes (Fig. 1). Even though the combined inhibition of *StpsPPase* and *StNTT* did not lead to a complete loss of gene expression, transcripts were reduced to a very low level compared with the wild type. The dry weights of mature *StpsPPase–StNTT*-inhibited potato tubers were low, ranging between 8% and 13% of FW (Table 1), which was within the same range as for the *StpsPPase*-inhibited lines and somewhat lower than for the *StNTT*-inhibited lines. The double-gene-inhibited tubers also had a very low starch content of 2–3% of FW (Table 1). These were within the same range as for the *StpsPPase*-inhibited tubers, but were, again, lower than that seen in the *StNTT*-inhibited tubers. An exception was line 753, which had a starch content of 6% of FW. The starch granules in the *StpsPPase–StNTT*-inhibited tubers were few compared with the wild type (Fig. 3F, H), being small spheres most with a size of ~15 µm (Fig. 3E). The *StpsPPase–StNTT*-inhibited tubers also had the same low amylose content as the *StpsPPase* single-gene-inhibited tubers, ranging between 3% and 5% amylose of total starch (Table 1).

### Tuber number and total tuber yield are differently affected in *StpsPPase–StNTT* simultaneously inhibited lines compared with single-gene inhibition

The number of tubers produced by lines 729, 753, and 764, with *StpsPPase* and *StNTT* simultaneous inhibition, was dissimilar to that produced by the single-gene-inhibited lines (Table 1; Fig. 4). Furthermore, all double-gene-inhibited lines displayed an average tuber weight that was considerably lower than that of the wild type (Table 1). Hence, in contrast to the single-gene-inhibited lines, the majority of the *StpsPPase*–*StNTT*-inhibited lines had an extremely low fresh weight tuber yield per plant: 5–25% (FW) that of the wild type. However, lines 754 and 767 had yields within the same range as the wild type.

All modified lines in the present study displayed phenotypic alteration. Tubers were elongated and often had one or two constrictions as well as adventitious tubers budding from the main tuber (Fig. 4) regardless of whether there was single-gene inhibition of either the *StpsPPase* or *StNTT* genes, or simultaneous down-regulation of both. No phenotypical changes were evident on the green biomass of any of the plants when compared with the wild type (data not shown).

### Inhibition of *StpsPPase* leads to decreased PPase activity and increased AGPase activity

Enzyme activities of PPase and AGPase were measured from total cellular potato tuber extracts in lines 606, 343, and 764 (Table 2). Only two-thirds of the average total PPase activity remained in the *StpsPPase*-inhibited tubers of 606 and 764, while the activity was statistically unaffected in the *StNTT* single-gene-inhibited line 343. Tubers from line 764 had, on average, a >50% increase in AGPase activity.

**Table 2.** PPase and AGPase enzyme activities measured on total cellular potato tuber extracts of *StpsPPase*- (606) and *StNTT*- (343) and *StpsPPase*+*StNTT*- (764) down-regulated potato lines

	AGPase (µmol g FW^–1^ min^–1^)	PPase (mmol g FW^–1^ min^–1^)
606	0.64 ± 0.12	0.24 ± 0.02*
343	0.44 ± 0.05	0.44 ± 0.04
764	0.84 ± 0.17*	0.24 ± 0.02*
Wt	0.37 ± 0.21	0.36 ± 0.03

Results presented are the mean ±SD of three biological and two technical replicates. Results marked with an asterisk differ significantly from the wild type (Wt) using one-way ANOVA, Tukey comparison method (*P*=0.05).

### Inhibition of *StpsPPase* and *StNTT* affects the amount of intermediate metabolites in the sugar metabolism and vitamin C pathway

**Fig. 5.**
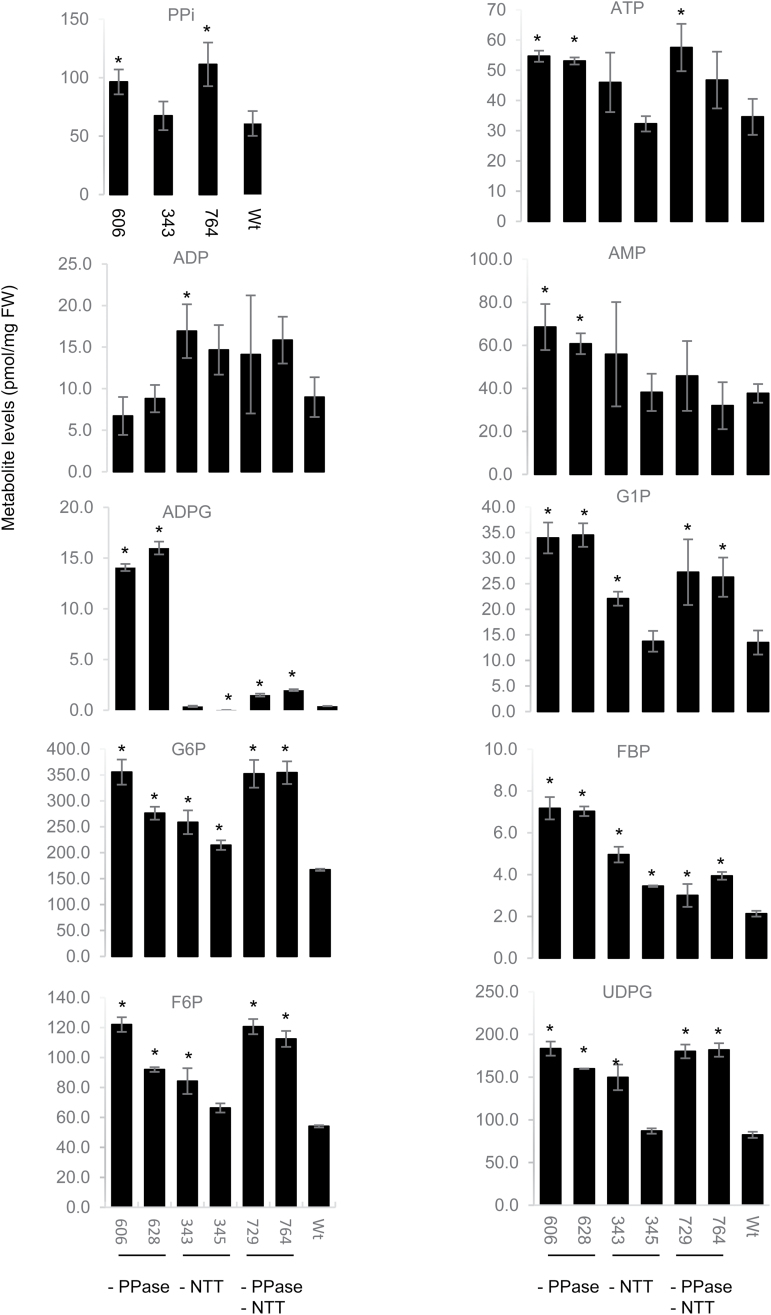
Amounts of metabolites in pmol mg FW^–1^ of lines 606 (*StpsPPase* inhibition); 345 (*StNTT* inhibition); 764 (*StpsPPase*+*StNTT*); and the wild type (Wt). PPi, pyrophosphate; ADPG, ADP-glucose; G1P, glucose-1-phosphate; G6P, glucose-6-phosphate; FBP, fructose-1,6-bisphosphate; F6P, fructose-6-phosphate; UDPG; UDP-glucose. Results presented are the mean ±SD of three biological replicates. Samples marked with an asterisk differ significantly from the wild type using one-way ANOVA, Tukey comparison method (*P*=0.05).

Metabolites were measured quantitatively in total cellular extracts of potato tubers (Fig. 5; Supplementary Table S2). Inhibition of *StpsPPase* resulted in a 60% increase in PPi, while it increased by 80% when *StpsPPase* and *StNTT* were inhibited simultaneously. Potato tubers also exhibited increased amounts of glucose and fructose (Supplementary Table S2), as well as of intermediates in the sugar and starch metabolic pathways (Fig. 5), namely fructose-1,6-biphosphate, fructose-6-phosphate, UDP-glucose, glucose-6-phosphate, and glucose-1-phosphate. The most elevated levels, compared with the wild type, occurred in tubers of the *StpsPPase*- and double-gene-inhibited lines. The amount of sucrose was lower in the transgenic tubers, with the exception of some of the double-gene-inhibited tubers where it was the same as in the wild type. A high amount of FBP was found in tubers of the *StpsPPase*-inhibited line, while in the double-gene-inhibited line the amounts were similar to those of the wild type (Fig. 5). The most striking observation was a 40-fold increase in ADP-glucose (ADPG) content in *StpsPPase*-inhibited tubers when compared with the wild type, while the *StpsPPase–StNTT*-double-inhibited tubers displayed a 5-fold increase (Fig. 5). No significant changes were seen in the amount of AMP, while ADP was increased in the *StNTT*-inhibited line compared with the wild type (Fig. 5). The amount of ATP was higher in the *StpsPPase*- and double-gene-inhibited tubers, while the *StNTT*-inhibited tubers exhibited no significant alterations compared with the wild type (Fig. 5). Hence, the ATP:ADP ratio was significantly lower in *StNTT*-inhibited tubers, at 2.2 and 2.7, while the ratio in *StpsPPase*-inhibited tubers was significantly higher, at 6.0 and 8.1 ,compared with 3.9 in the wild type (Tukey pairwise comparison). A higher accumulation of intermediates in the vitamin C biosynthetic pathway, galactonic acid-1,4-lactone and gulose, as well as mannose, was found in all studied lines (Supplementary Table S2). A significant increase of the organic acid glucaric acid-1,4-lactone was also detected (Supplementary Table S2); however, the biological significance of this metabolite in plants is unclear. Among the amino acids, increased amounts of arginine and decreased amounts of asparagine were most pronounced (Supplementary Table S2). Intermediates in the citric acid cycle were also affected, with aconitic acid, isocitric acid, and malic acid increasing, and succinic acid decreasing (Supplementary Table S2). Furthermore, there was a significant increase in the reducing sugar xylose, increased amounts of the starch degradation product maltose, and a decreased amount of glycerol-3-phosphate (Supplementary Table S2) in the *StpsPPase*- and *StNTT*-inhibited tubers, both individually and combined.

## Discussion

Potato lines, both with individually and simultaneously inhibited expression of a previously uncharacterized soluble plastidial *PPase* gene of *S. tuberosum* origin, *StpsPPase*, and an ATP/ADP translocator gene, *StNTT* (Tjaden *et al.*, 1998), were produced in order to study the influence of altered hydrolysis of the by-product PPi and adenylate supply. In microorganisms, such as, for example, *Saccharomyces cerevisiae*, mutations in PPase can be lethal (Perez-Castineira *et al.*, 2002; Serrano-Bueno *et al.*, 2013). Lower viability in tobacco plants may also occur if plastidial-localized PPase activity is absent (George *et al.*, 2010). Interestingly, in our study, almost complete inhibition of *StpsPPase* in the tuber sink tissue did not affect the general viability of the potato plants. It did, however, lead to a 1.5-fold decrease in total cellular PPase activity and a 1.6- and 1.8-fold increase in PPi content in potato tubers (Fig. 5; Table 2), confirming the PPi-hydrolysing function of the targeted putative PPase gene. PPi content has previously been reported to be 17 times higher in the cytosol than in the amyloplast of a potato cell (Farré *et al.*, 2001), hence our increase of PPi in a total potato tuber extract appears to indicate a significant accumulation of PPi in amyloplasts (Fig. 5).

The severe effect that *StpsPPase* inhibition had on starch accumulation, yielding tubers with only a tenth the starch content of the wild type (Table 1), may be explained by a loss of Pi needed for counter exchange lowering the import of glucose-6-phosphate into amyloplasts, perhaps in combination with a starch biosynthetic regulation derived from PPi accumulation in amyloplasts. The inhibition of *StNTT* also had a major negative effect on starch accumulation, although to a lesser extent than with *StpsPPase* inhibition. Their simultaneous inhibition gave rise to a low starch content similar to that caused by the single inhibition of *StpsPPase* (Table 1). Various models suggest how PPi formed in amyloplasts is dissipated. The increase in PPi and the deficiency in starch accumulation in tubers of our *StpsPPase*-inhibited lines indicate that, in accord with Farré *et al.* (2006), a plastidial export mechanism of PPi probably does not exist in potato amyloplasts, and that PPi pools in the cytosol and plastids are hypothetically independent of each other. Our results also support the hypothesis that StpsPPase is probably the only enzyme responsible for the PPi-hydrolysing activity in the amyloplast of potato tubers. In a study by George *et al.* (2010), where *NbPPase* was down-regulated in tobacco leaf chloroplasts, decreased amounts of transitory starch were found. These two studies together illustrate that PPase activity is vital for functional starch biosynthesis, independent of its localization in sink or source tissue.

Metabolic profiling revealed a surprisingly high amount of ADP-glucose in *StpsPPase*-inhibited potato tubers (Fig. 5). AGPase generating ADP-glucose from glucose-1-phosphate and ATP in dicots is generally regarded as the main rate-limiting step in starch biosynthesis since it is allosterically activated by 3-phosphoglyceric acid and inhibited by Pi (Sowokinos and Preiss, 1982). As a consequence of StpsPPase inhibition, only a small amount of Pi will be released in the amyloplasts, thus hampering any allosteric inhibition of AGPase. It has been demonstrated that increased PPi can negatively affect the forward biosynthetic reaction of AGPase (Amir and Cherry, 1972). However, while inhibition of *NbPPase* resulted in a 40% increase in AGPase activity (George *et al.*, 2010), unfortunately ADP-glucose could not be measured in their samples. In our study, AGPase activity was prominent in the *StpsPPase*-inhibited potato tubers, although only one line (764) had a statistically significant increased activity compared with the wild type (Table 2). The evident AGPase activity combined with the accumulation of PPi as well as ADP-glucose does not support the theory that PPi accumulation makes the AGPase forward reaction less favourable. We might speculate that if AGPase inhibition were hindered, the regulatory mechanisms and rate-limiting step might shift towards the next enzymatic step in the pathway, namely starch molecule initiation or elongation. In contrast, combining the inhibition of *StpsPPase* and *StNTT* did not yield the same high accumulation of ADP-glucose (Fig. 5). Hence, the malfunctioning ATP import could be considered the limiting step in these lines.

Even though starch synthesis, in all three gene inhibition approaches of this study, was decreased to an exceptionally low level, minor quantities of starch did in fact still accumulate in the tubers. An alternative route for ADP-glucose formation in dicotyledons has been suggested by studying ADP-glucose and transitory starch formation in *A. thaliana* leaf tissue lacking AGPase activity. From those studies, it was proposed that the cytosolic sucrose synthase (SuSy) could produce ADP-glucose from sucrose, to be transported into the chloroplast by an ADP-glucose translocator (Baroja-Fernández *et al.*, 2001; Bahaji *et al.*, 2011). However, such a transport protein has not yet been identified, and this theory remains controversial (Neuhaus *et al.*, 2005; Barratt *et al.*, 2009; Geigenberger, 2011). If a cytosolic synthesis of ADP-glucose and its plastidic import exists, we can conclude from our results that it is of very minor importance to total starch accumulation in heterotrophic organs such as potato tubers. It is more likely that a very low residual activity of the down-regulated enzymes was responsible for the small amounts of starch produced.

Amylose content of the residual starch decreased substantially in both *StpsPPase*-and *StNTT*-inhibited tubers (Table 1). However, it should be noted that in our study a colorimetric method based on amylose–iodine complex formation was used to quantify apparent amylose. However, amylose content commonly seems to be affected when levels of accumulated starch are altered; this is often explained by decreased ADP-glucose concentrations being less favourable for GBSS than SSS due to differences in *K*_m_ values for ADP-glucose (Baba *et al.*, 1990; Lloyd *et al.*, 1999). Small increases in amylopectin ratios have been found following the modified expression of various enzymes in starch biosynthesis, such as ATP/ADP translocator, AGPase, and ATP/ADP hydrolysing phosphatase (apyrase) (Tjaden *et al.*, 1998; Lloyd *et al.*, 1999; Riewe *et al.*, 2008). For plants to produce a really high ratio of amylopectin to amylose, the GBSS enzyme needs to be substantially inhibited (Kuipers *et al.*, 1994). Starch from the potato lines in our study were found to have an amylopectin content as high as that in potato lines with targeted GBSS inhibition (Kuipers *et al.*, 1994; Andersson *et al.*, 2003). The use of the *GBSS* promoter controlling the RNAi fragments in our study could, in theory, negatively affect the *GBSS* expression on a transcript level. However, we could see by qPCR that *GBSS* expression was not significantly altered in the lines, and, by extracting and studying starch bound proteins, the presence of GBSS was confirmed (Supplementary Fig. S2). Hence, no correlation could be found between GBSS, neither as transcripts nor as protein, and the reduction in amylose to amylopectin ratio. A more likely explanation for the reduced amylose synthesis could be that the change in granule size and morphology found in all lines could have affected GBSS granule inclusion, and thereby its activity in a granule context.

*StNTT* inhibition in potato has previously been described by Tjaden *et al.* (1998), where the constitutive *Cauliflower mosaic virus* 35S promoter was used for driving an antisense construct of the plastidial potato ADP/ATP transporter gene. The results from our tuber-directed inhibition of *StNTT* align with those of Tjaden *et al.* (1998), although the decrease in starch content and amylose yield was more pronounced in our study. This could have been due to a tuber-specific expression, or because we used the RNAi method, which may have inhibited gene expression more rather than the antisense technique.

The number of tubers increased in both *StpsPPase* and *StNTT* single-inhibited lines. Even though the tubers were considerably smaller than in the wild type, the tuber mass yield was higher in all single-gene-inhibited lines, except for one *StpsPPase* line that had a large variation in tuber yield among replicates (Table 1). All generated lines developed phenotypically altered tubers, being elongated and/or with additional buddings (Fig. 4). Such changes in tuber yield and phenotype have also been reported elsewhere (Müller-Röber *et al.*, 1992; Tjaden *et al.*, 1998; Hofvander *et al.*, 2004; Riewe *et al.*, 2008). Although different enzymes were targeted in those studies, a common observation was a decrease in tuber starch content and an increase in sugars such as glucose and fructose, indicating a possible effect of the starch/soluble sugar ratio. It has been postulated that excess sugars can lead to stress responses and adaptive mechanisms including hormonal, metabolic, and transcriptional changes (Price *et al.*, 2004; Weigelt *et al.*, 2009). It has also been suggested that low oxygen levels could stimulate potatoes to increase their surface area to volume ratio (Geigenberger, 2003; Riewe *et al.*, 2008), an adaptation that has been found in potatoes with altered adenylate content.

In comparison with single-gene-inhibited lines, the combined *StpsPPase*- and *StNTT*-inhibited lines did not yield the same consistent results concerning increased number and yield of tubers. In four of six lines, numbers of tubers were in the same range as for the wild-type line, but extremely small-sized tubers resulted in a FW tuber yield as low as 6% of that of the wild type in the most affected line (Table 1). Notably, the green biomass phenotype was unchanged, even though the starch accumulation and tuber formation were severely affected. Many studies (some mentioned above), in which enzyme activity in the amyloplast-located starch biosynthesis has been altered, have reported changes in starch content and composition. For example, manipulation of plastidial-localized PGM resulted in decreased starch content and an increase in sucrose and glucose, while no changes were seen in tuber morphology or yield (Tauberger *et al.*, 2000); an almost complete reduction of starch accumulation was found when AGPase was inhibited, accompanied by an increase in sucrose and glucose as well as tuber numbers and yield (Müller-Röber *et al.*, 1992). Even though these studied lines often underwent dramatic decreases in starch content, total tuber yield generally increased. The extremely low tuber yield we found in the majority of the simultaneously *StpsPPase*- and *StNTT*-inhibited lines is exceptional (Table 1).

In our studied lines, mannose, gulose, and galactonic acid-1,4-lactone all significantly increased (Supplementary Table S2). The increase in these metabolites could be a result of accumulating sugars stimulating an enhanced vitamin C pathway, since they are all intermediates in the ascorbate (vitamin C) biosynthetic pathway derived from glucose-6-phosphate. This is in line with a previous study on tomato fruits with an increased PPase activity yielding a higher ascorbic acid content, which also resulted in a decrease in starch accumulation and higher amounts of sucrose and glucose (Osorio *et al.*, 2013). It is also known that plants can increase vitamin C content as a consequence of stress (Gill and Tuteja, 2010; Wang *et al.*, 2012). The limited supply of ATP and increased amounts of PPi in the amyloplasts, and hence disturbed long-term energy storage, will lead to severe stress, so affecting tuber yield and development. Furthermore, it has been shown that stress can affect the amounts of hormones such as auxin and cytokinin, which have essential roles in the formation of tubers (Romanov *et al.*, 2000).

In summary, our results support the hypothesis that a transport mechanism of PPi between the plastid and cytosol is unlikely. We have shown that hydrolysis of PPi by the action of a PPase is of vital importance for functional starch accumulation in amyloplasts and that no PPi-consuming metabolic reactions, or any additional mechanism for splitting PPi, are present to a significant level. Due to a remarkably high accumulation of ADP-glucose in *StpsPPase*-inhibited potato tubers, we can speculate that an alternative regulatory mechanism exists elsewhere in the starch biosynthetic pathway if shifting from AGPase. However, further studies are needed to test this hypothesis and elucidate any underlying mechanism behind ADP-glucose accumulation. Our study has also shown that a functional ATP import and PPi hydrolysis are essential for tuber formation and development.

## Supplementary data

Supplementary data are available at *JXB* online.

Table S1. Primers used for PCR and qPCR analyses in this research.

Table S2. Relative quantities of metabolites in *StpsPPase*- and *StNTT*-down-regulated potato lines.

Fig. S1. Phylogenetic tree of pyrophosphorylase homologues.

Fig. S2. *GBSS* transcript levels and visualization of starch-bound proteins.

Supplementary Figure and Table

## Abbreviations:

AGPase: ADP-glucose pyrophosphorylase
GBSS: granule-bound starch synthase
GPT: glucose-6-phosphate/phosphate translocator
NTT: ATP/ADP translocator
PGM: phosphoglucomutase
Pi: inorganic phosphate
PPase: inorganic pyrophosphatase
PPi: pyrophosphate
SSS: soluble starch synthase
