# Supplementary material for: Inhibition of plastid PPase and NTT leads to major changes in starch and tuber formation in potato
Source: J Exp Bot. 2018 Mar 10;69(8):1913–24. doi: 10.1093/jxb/ery051 (PMC6018912; doi:10.1093/jxb/ery051)
Supplement: Supplementary Figure and Table [file ery051_suppl_supplementary-figure-and-table.pdf]

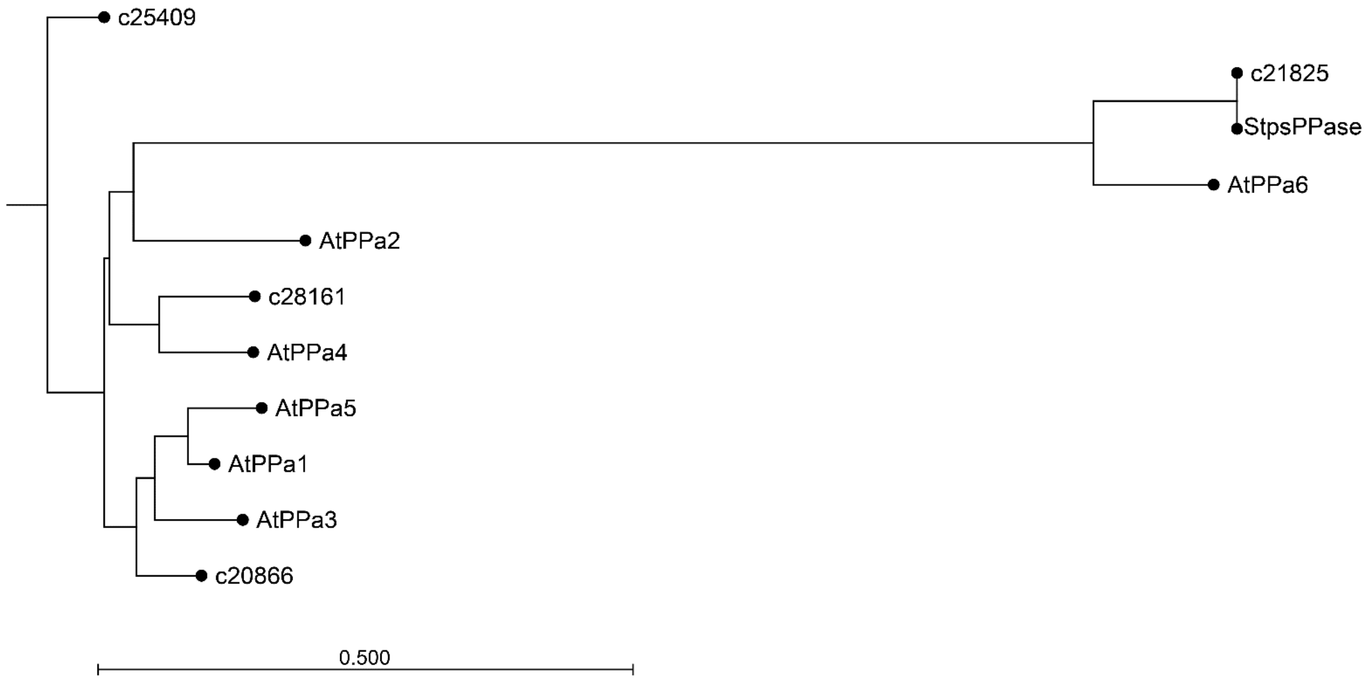

Figure S1. Phylogenetic tree of pyrophosphorylase homologs. Phylogenetic analysis based on aa sequences of pyrophosphorylase homologs from Arabidopsis (AtPPa1-6), aa sequence derived from tuber expressed contigs of potato variety Kuras (c20866. c21825, c25409 and c28161) (Hofvander *et al*., 2016) and the aa sequence deduced from the putative gene used in the present study (StpsPPase).

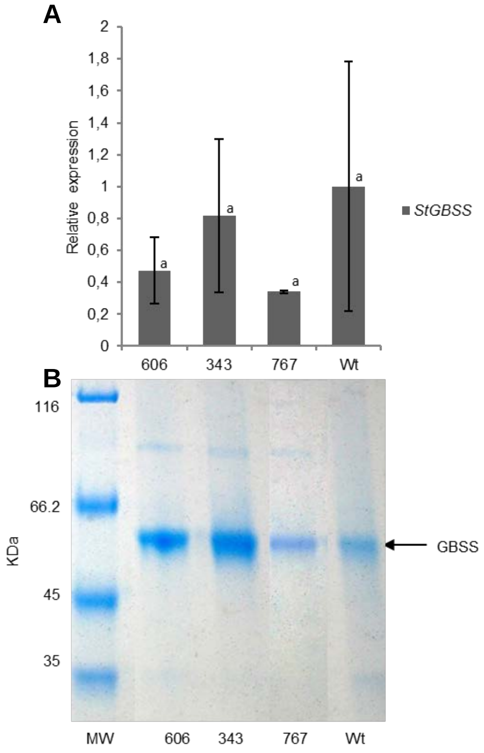

Fig. S2. *GBSS* transcript levels and visualization of starch bound proteins. **A.** Expression levels of *StGBSS* in lines 606 (*StpsPPase*-inhibition), 343 (*StNTT*-inhibition) and 767 (*StpsPPase+StNTT*-inhibition) and wild type (Wt) analyzed with real-time PCR. Expression levels of *StGBSS* were normalized to the reference gene actin. Expression level in wild type was set to one (1). Data are mean values (±SD) of three biological replicates. Samples sharing the same letters are not significantly different according to Tukey's test (P = 0.05).

B. Starch bound proteins separated on a 10% SDS-PAGE: 606 (*StpsPPase*-inhibition); 343 (*StNTT*-inhibition); 767 (*StpsPPase+StNTT* -inhibition); and wild type (Wt). Size marker (MW): Thermo Scientific™ Pierce™ Unstained Protein Molecular Weight Marker. GBSS protein (60 kDa) is marked with an arrow.

Table S1. Primers used for screening leaf tissue for transgene presence (NPTII) and *Agrobacterium* contamination (VirG) and primers used for analysis of gene expression using q-PCR (Actin, NTT, PPa, GBSS).

**Primer names**	**Primer sequence**
nptIIf	5`-CCTGTCATCTCACCTTGCTC-3´
nptIIr	5`-AGTCCCGCTCAGAAGAACTC-3`
VirG-	5´-GCCGGGGCGAGACCATAGG-3´
VirG+	5´- CGCACGCGCAAGGCAACC-3´
StActin-F	5´-ACAAGTGATGGCTGGAATAAGACTT-3´
StActinrev	5´-CCAGATGGTCAGGTGATTACCA-3´
qStnttF	5´-GTTTGGACTTGGAGCGAATG-3´
qStnttR	5´-TTTCAGGGAGATAGCCCAAC-3´
qStppa6.1F	5´-CAACAAGCCAGCAAACAAGG-3´
qStppa6.1R	5´-CAAGTGAAAGCTCACCAGCA-3´
StGBSS1f	5´-TTGCATAACTGGGATTGTGAATG-3´
StGBSS1r	5´-GACAGTGGTTATATCGTATTTGA-3´

Table S2. Relative quantities of metabolites in lines 606 and 628 (*StpsPPase*-inhibition); 343 and 345 (*StNTT*-inhibition); 718, 729, 753, 754, 764 and 767 (*StpsPPase+StNTT* -inhibition); and wild type (Wt). Wild type is set to 1. Significant values are marked in bold. n = 3.

Amino acids	Wt	606	628	343	345	718	729	753	754	764	767
Alanine	1±0,38	1,03±0,2	1,11±0,4	0,81±0,13	0,66±0,16	1,23±0,19	0,51±0,13	0,7±0,17	1,17±0,3	0,49±0,03	0,85±0,05
Arginine	1±0,03	**17,00±2,67**	**6,01±1,98**	**7,42±1,54**	**2,77±0,23**	**7,62±0,17**	**6,95±1,15**	7,04±6,23	**14,92±4,03**	**14,5±0,75**	**12,37±3,44**
Asparagine	1±0,04	**0,32±0,13**	**0,4±0,3**	**0,43±0,15**	**0,63±0,3**	**0,46±0,14**	**0,55±0,25**	**0,36±0,19**	**0,36±0,25**	**0,2±0,09**	**0,1±0,06**
Butyric acid, 4-amino	1±0,68	1,19±0,37	0,82±0,2	1,19±0,15	1,07±0,32	0,86±0,38	0,28±0,16	0,87±0,21	1,27±0,05	0,11±0,02	0,72±0,15
Cystine	1±0,29	0,97±0,1	**1,88±0,4**	0,72±0,1	0,97±0,23	**1,23±0,26**	1,41±0,34	1,14±0,18	0,94±0,33	0,65±0,03	0,79±0,2
Gluconic acid	1±0,29	0,68±0,11	0,73±0,13	1,05±0,52	0,76±0,21	0,82±0,33	0,81±0,06	**0,87±0,25**	0,67±0,14	1,17±0,1	0,75±0,13
Glutamic acid	1±0,25	0,87±0,15	1,05±0,31	1,05±0,16	1,44±0,17	0,93±0,32	1,07±0,08	1,31±0,32	0,85±0,4	**0,5±0,04**	0,68±0,22
Glutamine	1±0,28	1,38±0,74	0,73±0,35	0,60±0,25	1,18±0,85	1,14±0,24	0,58±0,3	**0,43±0,02**	1,07±0,18	0,6±0,3	0,55±0,12
Glycine	1±0,2	2,13±0,57	2,68±1,36	**2,03±0,37**	**2,16±0,26**	**2,73±0,27**	1,22±0,44	**1,53±0,38**	1,84±0,45	0,65±0,14	**1,48±0,24**
Histidine	1±0,41	**3,04±1,24**	2,99±2,59	**0,10±0,01**	**0,77±0,42**	**3,25±0,97**	2,01±0,88	1,02±0,12	2,09±1,3	0,7±0,4	0,78±0,3
Isoleucine	1±0,77	**2,68±0,23**	**3,2±1,72**	1,22±0,3	2,57±0,29	**3,17±1,38**	**2,57±0,99**	1,32±0,07	1,5±0,06	1,08±0,37	1,83±0,33
Lysine	1±0,64	**3,44±1**	4,2±3,52	0,75±0,37	**1,95±0,51**	4,31±2,34	**2,25±0,77**	1,34±0,28	2,47±1,1	0,77±0,33	1,38±0,47
Methionine	1±0,5	1,15±0,29	1,36±1	1,35±0,3	**2,04±0,85**	1,42±0,44	0,83±0,41	0,82±0,28	0,78±0,35	0,24±0,03	0,38±0,17
Ornithine	1±0,13	**5,09±1,78**	1,63±1,26	0,66±0,26	0,85±0,31	**3,51±0,88**	**1,95±0,38**	1,23±0,6	3,32±1,57	1,07±0,45	2,17±0,87
Phenylalanine	1±0,73	**3,17±0,12**	**2,49±0,77**	**3,10±0,52**	2,77±1,13	**3,3±0,5**	1,4±0,55	1,45±0,58	**2,29±0,29**	1,76±0,73	**3,26±0,4**
Proline	1±0,7	**1,77±0,44**	**2,63±0,79**	2,05±0,26	**3,04±0,35**	2,4±0,07	**1,88±0,5**	1,38±0,28	1,21±0,35	0,93±0,1	1,1±0,28
Purine, 6-benzylamino-	1±0,08	**2,27±0,36**	**6,13±1,15**	2,89±1,01	**4,87±1,33**	4,26±1,71	12,59±9,1	**3,51±0,72**	2,48±0,95	6,47±1,74	2,51±1,31
Putrescine	1±0,13	**4,83±0,91**	2,78±1,1	**2,31±0,33**	**2,11±0,41**	2,98±1,43	**2,21±0,12**	1,69±0,52	**2,38±0,1**	2,38±0,96	**2,7±0,73**
Pyroglutamic acid	1±0,2	0,84±0,26	0,59±0,24	0,77±0,09	1,03±0,43	0,79±0,24	0,54±0,18	0,53±0,15	0,84±0,52	**0,4±0,13**	**0,4±0,12**
Pyrroline-2-carboxylate	1±0,22	0,67±0,05	0,51±0,04	0,91±0,55	0,57±0,15	**0,35±0,07**	0,57±0,23	**0,31±0,06**	**0,33±0,14**	0,48±0,14	**0,3±0,08**

Serine	1±0,21	1,56±0,42	2,43±1,07	**2,21±0,24**	**3,13±0,51**	**3,17±0,37**	**1,93±0,59**	1,93±0,51	1,74±0,97	0,72±0,17	1,04±0,36
Threonine	1±0,27	**1,91±0,52**	**2,77±1,08**	**0,81±0,24**	**1,57±0,27**	**2,68±0,28**	**1,65±0,39**	1,19±0,25	1,43±0,61	0,54±0,15	1,1±0,35
Tryptophan	1±1,14	0,47±0,08	1,21±1,23	0,22±0,04	0,45±0,13	1,5±1,46	0,51±0,37	0,23±0,05	0,46±0,17	0,4±0,33	0,55±0,17
Tyramine	1±0,45	2,38±0,28	0,95±0,35	1,24±0,33	1,37±0,43	1,26±0,34	2,06±1,16	0,97±0,23	1,37±0,39	1,73±0,99	1,09±0,23
Tyrosine	1±1,03	1,77±0,21	5,14±4,76	0,79±0,29	1,86±0,49	4,05±3,04	**1,72±1,01**	1±0,1	1,53±0,36	0,9±0,64	1,75±0,73
Valine	1±0,37	**4,76±0,58**	**3,58±0,52**	**1,70±0,27**	**2,48±0,18**	**4,35±1,14**	**2,65±0,95**	**2,17±0,5**	**3,77±0,75**	1,57±0,46	**3,3±0,93**

**Organic acids**	**WT**	**606**	**628**	**343**	**345**	**718**	**729**	**753**	**754**	**764**	**767**
Aconitic acid	1±0,19	**2,73±0,21**	**5,87±1,03**	**1,86±0,53**	**3,11±1,15**	**8±2,39**	**5,49±0,41**	**2,99±0,8**	**2,68±0,75**	1,43±0,08	**2,74±0,85**
Adipic acid	1±0,09	**0,68±0,07**	0,65±0,29	0,87±0,54	1,35±0,66	0,99±0,35	1,09±0,43	1,06±0,31	0,75±0,74	**0,36±0,1**	0,65±0,34
Aspartic acid	1±0,03	1,17±0,27	1±0,31	**0,44±0,06**	**0,67±0,12**	1,35±0,65	0,92±0,22	**0,69±0,05**	**0,65±0,17**	**0,56±0,08**	**0,55±0,13**
Cinnamic acid, 4-hydroxy-	1±0,15	**1,36±0,16**	**2,64±0,53**	**1,43±0,1**	**1,77±0,16**	**1,68±0,16**	**2,01±0,38**	**1,53±0,26**	1,31±0,37	**1,68±0,14**	**1,19±0,15**
Eicosanoic acid methyl ester	1±0,18	1,23±0,05	**1,14±0,13**	1,11±0,03	1,17±0,09	1,1±0,02	1,08±0,07	0,99±0,03	1,11±0,02	1,01±0,07	1,13±0,1
Erythronic acid	1±0,28	**3,21±0,08**	**2,7±0,33**	**2,22±0,22**	**2,56±0,22**	2,67±0,85	**2,82±0,23**	**2,98±0,16**	2,13±0,9	1,49±0,13	1,82±1,28
Fumaric acid	1±0,17	1,53±0,36	1,42±0,17	**3,18±0,9**	3,71±2,92	3,93±1,79	0,96±0,19	1,4±0,62	0,93±0,28	0,73±0,05	1,05±0,15
Galactonic acid	1±0,29	0,68±0,11	0,73±0,13	1,05±0,52	0,76±0,21	0,82±0,33	0,81±0,06	**0,87±0,25**	0,67±0,14	1,17±0,1	0,75±0,13
Galactonic acid-1,4-lactone	1±0,24	**11,61±4,65**	**12,16±0,74**	5,15±3,75	3,25±3,13	**6,85±0,57**	**6,74±2,96**	6,32±7,07	**8,13±0,93**	36,13±21,91	**6,28±1,32**
Glucaric acid-1,4-lactone	1±0,22	**43,22±13,04**	**26,23±12,53**	**30,45±13,09**	22,34±21,29	14,35±8,49	**25,43±5,04**	**33,92±29,18**	**57,26±28,62**	**31,07±6,99**	**33,56±10,37**
Glyceric acid	1±0,21	**3,3±0,8**	**3,11±1,22**	1,42±0,1	**2,11±0,09**	**2,89±1,09**	1,71±0,44	**1,58±0,24**	1,56±0,19	0,71±0,15	1,39±0,27
Isocitric acid	1±0,22	**2,57±0,78**	1,62±0,65	**1,9±0,06**	2,64±1,06	**2,13±0,04**	1,4±0,41	**1,76±0,15**	**3,94±0,65**	1,38±0,41	**1,53±0,18**
Lactic acid dimer	1±0,23	1,01±0,07	0,9±0,08	1,02±0,05	1,03±0,13	0,91±0,03	1±0,03	0,94±0,03	1,06±0,14	0,99±0,07	1,09±0,19
Malic acid	1±0,36	**3,67±0,1**	**3,11±0,39**	**2,43±0,23**	**2,9±0,25**	**2,98±0,92**	**3,16±0,25**	**3,57±0,39**	2,81±1,47	1,71±0,17	**3,59±0,32**

Nicotinic acid	1±0,29	**1,56±0,41**	**2,6±0,24**	1,27±0,36	1,75±0,25	**1,82±0,12**	**1,84±0,54**	1,16±0,32	0,79±0,22	0,61±0	0,81±0,23
Nonadecanoic acid methyl ester	1±0,22	0,87±0,05	0,78±0,07	0,82±0,01	0,92±0,06	0,83±0,01	0,9±0,02	0,92±0,01	0,91±0,05	0,91±0,01	1,01±0,14
Octadecanoic acid	1±0,15	0,95±0,06	**0,9±0,15**	0,88±0,06	0,82±0,07	**0,76±0,04**	**0,88±0,11**	0,78±0,03	0,9±0,01	1,14±0,01	1,19±0,15
Phosphoric acid	1±0,21	**0,6±0,09**	**0,67±0,08**	0,63±0,03	0,78±0,03	**0,63±0,07**	0,77±0,05	0,92±0,12	0,6±0,08	0,67±0,06	**0,48±0,04**
Quinic acid	1±0,12	2,24±0,66	**2,9±0,26**	2,3±0,83	**3,08±0,64**	**2,35±0,58**	**4,05±0,31**	**3,09±0,78**	**2,2±0,33**	**4,78±0,04**	**2,56±0,61**
Succinic acid	1±0,19	**0,54±0,07**	**0,53±0,07**	**0,46±0**	**0,35±0,11**	0,47±0,18	**0,3±0,04**	**0,35±0,04**	**0,39±0,01**	**0,44±0,04**	**0,48±0,05**
Tetradecanoic acid methyl ester	1±0,17	0,9±0,05	0,79±0,06	0,87±0,01	0,9±0,05	0,84±0,02	0,88±0,03	0,88±0,03	0,89±0,05	0,92±0,01	1±0,12
Threonic acid	1±0,05	1,08±0,23	0,92±0,27	**0,49±0,07**	**0,66±0,1**	1,2±0,56	0,84±0,17	**0,67±0,05**	**0,64±0,15**	**0,54±0,08**	**0,55±0,12**
Triacontanoic acid methyl ester	1±0,13	1,04±0,06	0,91±0,07	0,86±0,02	1,04±0,1	0,87±0,05	0,96±0	0,98±0,09	1,05±0,04	1,1±0,02	**1,16±0,14**

**Sugars**	**Wt**	**606**	**628**	**718**	**729**	**753**	**754**	**764**	**767**
Erythrose	1±0,23	1,01±0,07	0,9±0,08	0,91±0,03	1±0,03	0,94±0,03	1,06±0,14	0,99±0,07	1,09±0,19
Fructose	1±0,21	1,22±0,64	4,21±3,29	4,3±2,36	4,07±4,06	2,84±2,06	1,09±0,24	**2,77±0,64**	**1,74±0,25**
Galactinol	1±0,24	**0,39±0,08**	1,01±0,55	0,66±0,51	2,9±1,1	0,76±0,32	**0,35±0,1**	**0,45±0,01**	0,85±0,32
Glucose	1±0,24	**1,66±0,12**	1,37±0,14	1,24±0,03	1,42±0,12	**1,77±0,13**	**2,03±0,11**	**2,1±0,11**	**2,38±0,35**
Glycerol	1±0,2	0,99±0,04	0,91±0,09	0,91±0,01	0,93±0,05	0,99±0,04	0,96±0,03	0,89±0,03	0,97±0,14
Glycerol-3-phosphate	1±0,1	**0,32±0,05**	**0,34±0,08**	**0,27±0,04**	**0,46±0,08**	**0,49±0,14**	**0,37±0,15**	**0,4±0,11**	**0,2±0,04**
Gulose	1±0,25	**2,96±0,24**	**5,61±0,78**	**7,59±2,18**	**5,81±0,23**	**3,42±0,83**	2,54±1,2	1,33±0,32	2,74±1,27
Kestose	1±0,37	4,86±2,01	5,21±3,83	4,43±3,09	2,63±1,63	5,15±2,64	**5,19±0,42**	**5,27±2,49**	**6,32±1,33**
Maltose	1±0,74	**11,84±2,63**	**9,6±3,64**	8,23±3,5	**7,99±2,14**	6,17±4,44	**9,96±3,07**	**14,69±0,36**	**10,66±2,33**
Mannitol	1±0,17	**2,27±0,11**	**3,78±0,42**	**4,77±1,04**	**3,68±0,19**	**2,34±0,47**	1,94±0,62	1,22±0,12	**2,19±0,61**
Mannose	1±0,25	**2,96±0,24**	**5,61±0,78**	**7,59±2,18**	**5,81±0,23**	**3,42±0,83**	2,54±1,2	1,33±0,32	2,74±1,27

Raffinose	1±0,37	4,86±2,01	5,21±3,83	4,43±3,09	2,63±1,63	5,15±2,64	**5,19±0,42**	**5,27±2,49**	**6,32±1,33**
Rhamnose	1±0,26	1,02±0,08	**1,7±0,33**	**1,67±0,42**	1,93±0,65	1,29±0,21	0,93±0,25	1,04±0,14	0,89±0,11
Sucrose	1±0,06	**0,67±0,05**	**0,55±0,06**	**0,46±0,08**	**0,62±0,06**	0,82±0,08	1±0,06	1,05±0,04	1,13±0,15
Tagatose	1±0,15	0,91±0,06	0,97±0,1	1,05±0,07	1,1±0,01	1,06±0,05	1,01±0,09	0,9±0,02	1,1±0,1
Xylose	1±0,3	**4,99±1,02**	2,66±1,21	**3,07±0,22**	4,94±2,44	2,45±1,74	3,6±1,63	**9,2±1,25**	**5,16±1,04**
Xylulose	1±0,23	**0,84±0,15**	1,01±0,31	0,88±0,3	1,03±0,09	1,23±0,34	0,76±0,32	**0,5±0,03**	0,64±0,2

**Others**	**Wt**	**606**	**628**	**343**	**345**	**718**	**729**	**753**	**754**	**764**	**767**
Arabitol	1±0,08	0,99±0,07	0,95±0,03	1,01±0,03	0,98±0	0,97±0,01	1,01±0,03	0,97±0,05	1±0,07	1±0,04	0,99±0
Calystegine A3	1±0,08	**0,77±0,09**	0,7±0,28	0,89±0,52	1,4±0,65	1,09±0,39	1,15±0,47	1,1±0,29	0,76±0,73	**0,4±0,12**	0,71±0,35
Calystegine B2	1±0,06	**0,55±0,12**	0,89±0,38	0,6±0,43	1,32±0,65	1,02±0,33	1,36±0,57	1,12±0,25	0,58±0,56	**0,53±0,09**	0,56±0,31
Glucosone	1±0,2	**1,85±0,29**	**1,78±0,51**	**2,34±0,27**	**2,05±0,61**	1,4±0,6	**1,45±0,17**	**1,67±0,31**	**1,91±0,09**	**1,8±0,09**	1,43±0,28
Hexadecanoic acid methyl ester	1±0,17	1,04±0,04	0,87±0,09	0,96±0,04	1,01±0,07	0,9±0,03	1,01±0,04	1,04±0,06	1,15±0,03	1,19±0,02	1,22±0,09
Naringenin	1±0,11	2,42±0,76	**3,3±0,36**	2,61±1,01	**3,51±0,8**	**2,65±0,71**	**4,76±0,24**	**3,26±0,83**	**2,27±0,36**	**5,53±0,14**	**2,72±0,67**
Stigmasterol	1±0,11	**1,17±0,07**	**1,12±0,09**	1,04±0,01	1,2±0,11	1,06±0,07	1,04±0,04	1,02±0,1	1,06±0,04	1,05±0,07	1,13±0,15
Tocopherol	1±0,14	1,15±0,4	**1,54±0,14**	0,57±0,17	1,06±0,42	1,3±0,3	**1,9±0,23**	1,28±0,54	0,9±0,35	**3,83±0,75**	**1,9±0,32**
Urea	1±0,1	0,92±0,08	**0,77±0,13**	1,07±0,24	1,35±0,48	0,91±0,17	0,94±0,1	**0,85±0,12**	**0,59±0,12**	**0,8±0,15**	0,96±0,12
